# High-quality draft genome of *Lactiplantibacillus plantarum* strain APC2688 isolated from human feces

**DOI:** 10.1128/mra.00641-24

**Published:** 2024-10-14

**Authors:** Benjamin Valderrama, Isabelle Daly, Eoin Gunnigle, Mary C. Rea, John Kenny, Mairead Coakley, John F. Cryan, Gerard Clarke, Jatin Nagpal

**Affiliations:** 1 APC Microbiome Ireland, University College Cork, Cork, Ireland; 2 Department of Anatomy and Neuroscience, University College Cork, Cork, Ireland; 3 Teagasc Food Research Centre, Moorepark, Fermoy, Cork, Ireland; 4 Department of Psychiatry & Neurobehavioural Sciences, University College Cork, Cork, Ireland; 5 Department of Pharmacology & Therapeutics, School of Medicine and School of Pharmacy, University College Cork, Cork, Ireland; University of Maryland School of Medicine, Baltimore, Maryland, USA

**Keywords:** *Lactiplantibacillus plantarum*, gut microbiota, probiotics, gut-brain axis

## Abstract

*Lactiplantibacillus plantarum* is a commensal bacterial species often found within the human gut. To expand our knowledge about this human-associated taxon with potential probiotic properties, here we present the draft genome sequence of *Lactiplantibacillus plantarum* strain APC2688, isolated from a human fecal sample.

## ANNOUNCEMENT

*Lactiplantibacillus plantarum* is a bacterial species often associated with benefits for host health due to the metabolites it produces during food fermentation as a gut commensal (1). Previous studies showed the potential of some *L. plantarum* strains to target the gut-brain axis (2, 3). Therefore, the genomic characterization of *L. plantarum* strains is the first step in the search for probiotic bacteria. In this context, we introduce the high-quality draft genome of *L. plantarum* APC2688. The strain was isolated from a fecal sample of a healthy participant of a study conducted in APC Microbiome Ireland. The study was approved by the Clinical Research Ethics Committee of the Cork Teaching Hospitals (Study number APC055) and was conducted in accordance with the ICH Guidelines on Good Clinical Practice, and the Declaration of Helsinki. All participants provided written informed consent. *L. plantarum* APC2688 was isolated from a fresh fecal sample on Lactobacillus selective (LBS) agar containing 50 units/mL nystatin in November 2015. A single colony was sub-cultured in mMRS broth for 24–48 hours and then stored in glycerol (30%) at −80°C. *L. plantarum* APC2688 was grown from a glycerol stock on mMRS agar for 48 hours under anaerobic conditions in PLAS-LABS Simplicity 888 Automatic Atmosphere Chamber (N2: 80%, H2: 10%, CO2: 10%), at 37°C.

A single colony from the agar plate was inoculated into mMRS broth, grown anaerobically for 24–48 hours, and DNA was extracted using the Qiagen PowerFecal Pro DNA kit (Qiagen) according to the manufacturer’s guidelines. Library preparation and sequencing were performed by SeqBiome Ltd. (Cork, Ireland). Libraries were prepared using Nextera XT DNA Library Preparation Kit. Library preparation steps included DNA fragmentation, adapter ligation, amplification, and size selection steps. *L. plantarum* APC2688 genome was sequenced using Illumina NextSeq2000 (2  ×  150 bp), producing 5,599,109 paired reads. The quality of the raw sequencing was inspected with FastQC v0.11.9 (4) and Kneaddata v0.9.0 (https://github.com/biobakery/kneaddata) was used for quality-control reads. Trimmomatic v0.33 (5) was used for quality filtering with the following parameters: ILLUMINACLIP:/NexteraPE.fa:2:30:10:8:True SLIDINGWINDOW:5:25 MINLEN:110 TRAILING:25 AVGQUAL:25. Genome assembly was performed using Unicycler v0.5.0 (6). The completeness and the contamination percentage of the assembled genome sequence were determined using CheckM v1.1.3 (7), and GTDB-Tk v1.3.0 (8) was used for taxonomic classification of the genome. Genomic assembly was annotated with NCBI’s prokaryotic genome annotation pipeline (PGAP) v6.7 (9). Default parameters were used except where otherwise noted.

The genome of *L. plantarum* APC2688 is 3,432,914 bp long and was assembled in 115 contigs with a N_50_ of 107,233 and a coverage of 3.3×. The completeness of the genome was estimated as 99.38% with 2.78% contamination. Therefore, according to the MISAG (minimum information about a single amplified genome) standard, the genome here introduced is considered a high-quality genome draft (10). The genomic features of the strain are summarized in Table 1.

**TABLE 1:** Genome content statistics of *Lactiplantibacillus plantarum* APC2688

Characteristic	*L. plantarum* APC2688
Total bases (bp)	3,432,914
Estimate of completeness	99.38%
Contigs	115
N_50_	107,233
Coverage	3.3×
GC (%)	44.50
Genes	3,330
CDSs	3,258
Complete rRNAs (5S, 16S, 23S)	2
tRNA	66

## Data Availability

The genomic assembly of *L. plantarum* APC2688 was deposited in the GenBank under the accession number GCA_040183155.1. The raw reads were deposited in the Sequence Read Archive (SRA) under the accession number SRR29342813.

## References

[1] Echegaray N, Yilmaz B, Sharma H, Kumar M, Pateiro M, Ozogul F, Lorenzo JM. 2023. A novel approach to Lactiplantibacillus plantarum: from probiotic properties to the omics insights. Microbiol Res 268:127289. doi:10.1016/j.micres.2022.127289 36571922

[2] Bravo JA, Forsythe P, Chew MV, Escaravage E, Savignac HM, Dinan TG, Bienenstock J, Cryan JF. 2011. Ingestion of Lactobacillus strain regulates emotional behavior and central GABA receptor expression in a mouse via the vagus nerve. Proc Natl Acad Sci U S A 108:16050–16055. doi:10.1073/pnas.1102999108 21876150 PMC3179073

[3] Davis DJ, Doerr HM, Grzelak AK, Busi SB, Jasarevic E, Ericsson AC, Bryda EC. 2016. Lactobacillus plantarum attenuates anxiety-related behavior and protects against stress-induced dysbiosis in adult zebrafish. Sci Rep 6:33726. doi:10.1038/srep33726 27641717 PMC5027381

[4] Andrews S. 2010. FASTQC. A quality control tool for high throughput sequence data

[5] Bolger AM, Lohse M, Usadel B. 2014. Trimmomatic: a flexible trimmer for Illumina sequence data. Bioinformatics 30:2114–2120. doi:10.1093/bioinformatics/btu170 24695404 PMC4103590

[6] Wick RR, Judd LM, Gorrie CL, Holt KE. 2017. Unicycler: resolving bacterial genome assemblies from short and long sequencing reads. PLOS Comput Biol 13:e1005595. doi:10.1371/journal.pcbi.1005595 28594827 PMC5481147

[7] Parks DH, Imelfort M, Skennerton CT, Hugenholtz P, Tyson GW. 2015. CheckM: assessing the quality of microbial genomes recovered from isolates, single cells, and metagenomes. Genome Res 25:1043–1055. doi:10.1101/gr.186072.114 25977477 PMC4484387

[8] Parks DH, Chuvochina M, Rinke C, Mussig AJ, Chaumeil P-A, Hugenholtz P. 2022. GTDB: an ongoing census of bacterial and archaeal diversity through a phylogenetically consistent, rank normalized and complete genome-based taxonomy. Nucleic Acids Res 50:D785–D794. doi:10.1093/nar/gkab776 34520557 PMC8728215

[9] Tatusova T, DiCuccio M, Badretdin A, Chetvernin V, Nawrocki EP, Zaslavsky L, Lomsadze A, Pruitt KD, Borodovsky M, Ostell J. 2016. NCBI prokaryotic genome annotation pipeline. Nucleic Acids Res 44:6614–6624. doi:10.1093/nar/gkw569 27342282 PMC5001611

[10] Bowers RM, Kyrpides NC, Stepanauskas R, Harmon-Smith M, Doud D, Reddy TBK, Schulz F, Jarett J, Rivers AR, Eloe-Fadrosh EA, et al.. 2017. Minimum information about a single amplified genome (MISAG) and a metagenome-assembled genome (MIMAG) of bacteria and archaea. Nat Biotechnol 35:725–731. doi:10.1038/nbt.3893 28787424 PMC6436528

